# A 64k pixel CMOS-DEPFET module for the soft X-rays DSSC imager operating at MHz-frame rates

**DOI:** 10.1038/s41598-023-38508-9

**Published:** 2023-07-21

**Authors:** Stefano Maffessanti, Karsten Hansen, Stefan Aschauer, Andrea Castoldi, Florian Erdinger, Carlo Fiorini, Peter Fischer, Pradeep Kalavakuru, Helmut Klär, Massimo Manghisoni, Christian Reckleben, Lothar Strüder, Matteo Porro

**Affiliations:** 1 Deutsches Elektronen-Synchrotron DESY, Notkestr. 85, 22607 Hamburg, Germany; 2 PNSensor GmbH, 81739 Munich, Germany; 3 Dipartimento di Elettronica, Informazione e Bioingegneria, Politecnico di Milano, 20133 Milan, Italy; 4 Istituto Nazionale di Fisica Nucleare, Sezione di Milano, 20133 Milan, Italy; 5 EXTOLL GmbH, 68159 Mannheim, Germany; 6 Institute for Computer Engineering (ZITI), Heidelberg University, 69120 Heidelberg, Germany; 7 European XFEL, Holzkoppel 4, 22869 Schenefeld, Germany; 8 Dipartimento di Ingegneria e Scienze Applicate, Università di Bergamo, 24044 Dalmine, Italy; 9 Istituto Nazionale di Fisica Nucleare, Sezione di Pavia, 27100 Pavia, Italy; 10 University of Siegen, 51228 Siegen, Germany; 11 Department of Molecular Sciences and Nanosystems, Ca’ Foscari University of Venice, 30172 Venezia, Italy

**Keywords:** Electrical and electronic engineering, Techniques and instrumentation

## Abstract

The 64k pixel DEPFET module is the key sensitive component of the DEPFET Sensor with Signal Compression (DSSC), a large area 2D hybrid detector for capturing and measuring soft X-rays at the European XFEL. The final 1-megapixel camera has to detect photons with energies between $250\,\text{eV}$ and $6\,\text{keV}$, and must provide a peak frame rate of $4.5\,\text{MHz}$ to cope with the unique bunch structure of the European XFEL. This work summarizes the functionalities and properties of the first modules assembled with full-format CMOS-DEPFET arrays, featuring $512\,\times \,128$ hexagonally-shaped pixels with a side length of 136 μm. The pixel sensors utilize the DEPFET technology to realize an extremely low input capacitance for excellent energy resolution and, at the same time, an intrinsic capability of signal compression without any gain switching. Each pixel of the readout ASIC includes a DEPFET-bias current cancellation circuitry, a trapezoidal-shaping filter, a 9-bit ADC and a 800-word long digital memory. The trimming, calibration and final characterization were performed in a laboratory test-bench at DESY. All detector features are assessed at $18\,^{\circ }\text{C}$. An outstanding equivalent noise charge of $9.8$e^−^rms is achieved at ﻿1.1-MHz frame rate and gain of 26.8 Analog-to-Digital Unit per keV ($\,\text {ADU}/\text{keV}$). At $4.5\,\text{MHz}$ and $3.1\,\,\text {ADU}/\text{keV}$, a noise of $25.5$ e^−^rms and a dynamic range of $26\,\text{k}\text {e}^{-}$ are obtained. The highest dynamic range of $1.345\,\text{M}\text {e}^{-}$ is reached at $2.25\,\text{MHz}$ and $1.6\,\text {ADU}/\text{keV}$. These values can fulfill the specification of the DSSC project.

## Introduction

The European XFEL (EuXFEL) is an X-ray Free Electron Laser source, where up to 2700 extremely brilliant X-ray pulses of a single bunch train at $4.5\,\text{MHz}$ are repeated every $100\,\text{ms}$^1^. Its unique bunch scheme poses big design challenges for the imaging detector development. Three 1-megapixel detector types have been specifically designed with different concepts to cope with the required X-ray energy range, peak frame rate and dynamic range.

The Large Pixel Detector (LPD)^2^ has square pixels of ${500}$-μm size, and was designed to be operated in the energy range between $5$ and $20\,\text{keV}$. Its pixel electronics features a charge sensitive amplifier (CSA) with three gain stages and a 512-cell analogue memory per stage operated in parallel. The digitization is executed during the train gaps thanks to an on-chip column-level analog-to-digital converter (ADC). The convenient gain path is selected off-chip in order to achieve the maximum dynamic range. The detector is part of the Femtosecond X-ray Experiments (FXE) scientific instrument at EuXFEL^3^.

The Adaptive Gain Integrating Pixel Detector (AGIPD)^4^ targets the same energy range as the LPD, but offers a spatial resolution of $200$ μm. It features a CSA with three gains dynamically selected depending on the CSA output. A correlated double sampling (CDS) stage removes the reset noise, and its output is stored in a 352-cell analogue memory. Its analog data is subsequently digitized by off-chip ADCs. The AGIPD is part of the Single Particles, Clusters, and Biomolecules & Serial Femtosecond Crystallography (SPB/SFX)^5^ and of the Materials Imaging and Dynamics (MID)^6^ Instruments.

The Depleted Field Effect Transistor (DEPFET) Sensor with Signal Compression (DSSC) is targeting the soft X-ray range between $250\,\text{eV}$ and $6\,\text{keV}$. A first camera is based on passive miniaturized silicon drift detector (mini-SDD) cells of hexagonal shape with a side length of $136$ μm^7^, corresponding to an equal-area diameter of $247$ μm. The readout chain of each pixel comprises a CSA, a time-variant filter with trapezoidal weighting function, a 9-bit ADC with gain-and offset-trimming capability, and a SRAM with a storage capacity of 800 samples. Therewith, the DSSC detector not only offers the deepest storage capacity among the three detector versions, but is also unique with its per-pixel digitizer approach. Another unique feature concerns the power-down capability of unused analog and mixed-signal blocks during memory readout within inter-train gaps. In this way, the in-vacuum power dissipation is drastically reduced to $149\,\text{W}$ in contrast to the AGIPD ($550\,\text{W}$). The power consumption of the 1-megapixel detector comprising the outside-vacuum electronics is $263\,\text{W}$ compared to the AGIPD ($1.2\,\text{kW}$^4^) and LPD ($12\,\text{kW}$^8^). The gain of the signal processing chain can be adjusted in the CSA, in the filter and in the ADC, so that the current version of the DSSC imager with passive mini-SDD sensor covers the entire energy range with a gain granularity below one percent^7^. The imager reached an equivalent noise charge (ENC) of about $60\,\text {e}^{-}\text {rms}$ at the peak frame rate of $4.5\,\text{MHz}$, where the linear dynamic range is limited to maximal 9 bit. The camera was commissioned and is in use at the Spectroscopy and Coherent Scattering (SCS)^9^ and Small Quantum Systems (SQS) soft X-ray instruments at EuXFEL.

Other detectors targeting the soft X-ray regime well below $1\,\text{keV}$ are also based on the hybrid technology or originate from the class of charge-coupled devices (CCD) and monolithic CMOS imagers. They offer a higher spatial resolution but are limited in frame rates. To improve the performance at low X-ray energies, a novel CCD readout, called single electron sensitive readout (SiSeRO), is being developed at the MIT Lincoln Laboratory^10^. It features a floating gate amplifier composed by a MOSFET transistor with an internal gate used to read out the charge collected by the CCD matrix, and is based on the repetitive non-destructive readout (RNDR) DEPFET concept^11^. The authors of the paper could obtain a noise performance of $15\,\text {e}^{-}\text {rms}$ at $500\,\text{kpixel}/{\rm s}$, corresponding to a frame rate of about $2\,\text{Hz}$ of their readout concept. As hybrid candidate, the MÖNCH detector reached an ENC of about $40\,\text {e}^{-}\text {rms}$ at $3\,\text {kfps}$ for a unique ${25}$-μm pixel pitch^12^. The pnCCD detector with ${75}$-μm pixel pitch is part of the SQS instrument at EuXFEL and can be operated up to 100-Hz${\text{Hz}}$ frame rates. In user experiments, an ENC around $10\,\text {e}^{-}\text {rms}$ was achieved^13^. A monolithic example is the soft X-ray CMOS image sensor (sxCMOS), which is based on low-oxygen concentration Czochralski-grown silicon wafers and backside thinned to $45$ μm. With a pixel pitch of $22.4$ μm, the imager achieved $8.1\,\text {e}^{-}\text {rms}$ noise level at a speed of $450\,\text{Hz}$^14^. Except sxCMOS, all aforesaid cameras utilize thick high-resistivity wafer substrates for photon absorption, enabling their efficient use also at higher photon energies, whereas classical CMOS imagers make use of thin epitaxial layers. Recently, a sufficient quantum efficiency in soft X-ray domain was demonstrated for backside-illuminated imagers. Utilizing the $10$ μm thick epi-layer of a commercial 180-nm CMOS technology and external post-processing of its backside, the ${27}$-μm pixel pitch Percival detector obtained a maximum frame rate and minimum ENC of about $83\,\text{Hz}$ and $16\,\text {e}^{-}\text {rms}$, respectively^15^. Moreover, Desjardins et al.^16^, used a fully commercial CMOS imager (GSENSE 400BSI-GP) with ${4}$-μm epi-layer and ${11}$-μm pixel size for experiments at the soft X-ray branch of the metrologie beamline at SOLEIL synchrotron. They achieved a minimal ENC of $6\,\text {e}^{-}\text {rms}$ at a frame rate of $24\,\text{Hz}$. Fully-depleted CMOS imager approaches based on thinned and post-processed high-resistivity substrates also exist for soft X-ray applications^17,18^. In order to comply with the targeted high sensitivity and frame rate, the pixel electronics of the ${50}$-μm pixel pitch ePixM detector is limited to nine transistors, and the digitization is shifted to a bump-bonded ADC tier. They strive for a frame rate and noise of $24\,\text{kHz}$ and $11\,\text {e}^{-}\text {rms}$, respectively.

The second DSSC camera addresses the challenge of low noise at high frame rates and is currently under construction. The new camera utilizes DEPFET pixel arrays in a fully customized double-sided 350-nm 2-poly 3-metal high-voltage CMOS process^19^. This active pixel sensor is based on $725$ μm thick high-resistivity Si wafers extending not only the quantum efficiency far beyond the soft X-ray regime but also acting as radiation shield for the electronics layers behind the fully depleted Si bulk. The sensor combines an extremely low input capacitance for excellent energy resolution with an intrinsic capability of signal compression without any gain switching procedures in the analog front end. Signal charges are collected on an embedded internal gate implantation located underneath the DEPFET gate of the transistor for drain-current modulation. Its doping profile and shape causes the required non-linear response and extends the dynamic range maintaining the required single-photon resolution in the linear gain region. Other benefits of the DEPFET approach concern its capability of pixel-size shrinkage below $30$ μm^19^ and of non-destructive readout to reach sub-electron noise performance^11^. These features underline the flexibility and future potential of this detector concept. An overview of the DEPFET devices development is detailed by Andricek et al.^20^ The authors present the DEPFET applications dividing the device types in two subgroups: standard devices, used in high energy physics experiments and astrophysics, and non-standard devices. An example of the latter are the DEPFET with signal compression used in transmission electron microscopy and the RNDR recently proposed for the search of dark matter.

This work summarizes the functionalities and properties of the DSSC prototype modules, equipped with full-format CMOS-DEPFET sensors, for the first time. In “Camera and methods” section, a summary on the main building blocks of the camera-head electronics is given. We describe the trimming-relevant functionalities of the front-end electronics and introduce the experimental methods for performance verification. The “Results” section presents the laboratory test results of the individual key parameters measured at DESY. In particular, the sensor-pixel leakage current, the DEPFET-quiescent current, the electronics channel gain and offset in the primary linear gain region, the gain compression, and the total noise will be treated. The appraisal of the obtained results and comparison to other detectors are carried out in “Discussion and perspective” section.

## Camera and methods

### Camera head and periphery

The ladder camera operates a matrix of $512\,\times \,128$ DEPFET pixels and represents the smallest independent building block of the megapixel camera. Therefore, we expect that the obtained results from the ladder prototypes are also representative for the full camera. Except for some DEPFET-related functions, the whole electronics shares the same design of the mini-SDD camera version. For a more detailed summary, we refer the readers to the mini-SDD review^7^. In-depth descriptions on individual functions of all sub-assemblies can be found in the corresponding citations mentioned below.

The ladder camera consists of a focal-plane module (FPM), four regulator boards (RB), a central I/O board (IOB), and a module-interconnection board (MIB). The FPM consists of a metal frame, main board, heat spreader, and sixteen application-specific integrated circuits (ASIC) mounted on two CMOS-DEPFET sensors^21^. Its main board distributes the ASIC- and sensor-related supply voltages as well as their clock and control signals. The RBs provide the permanent and cycled supplies for the ASICs and main board^22^. On-board gate drivers (GD) generate pulses to remove the signal charges collected by the internal gates of the DEPFET matrix. The IOB concentrates the data of the ASICs to four ${3.125}{\text{-}}{\text{Gbit}/{\rm s}}$ links, controls the RBs, and provides the cycling of the DEPFET source and gate voltages^23^. The MIB provides the control signals for the GDs by on-board module amplifiers (MA)^22^, and collects all power supply, clock and data channels. A single flex cable connects the ladder electronics to the peripheral patch-panel electronics, which is located outside the vacuum chamber. Its mother board represents the interface to external power supplies, and hosts the patch-panel transceiver (PPT) and safety-interlock board (SIB). The PPT reads the data streams of four IOBs (in our setup only a single ladder is connected), concentrates them in a suitable data format to four optical 10 Gigabit-Ethernet links, and supports a machine-synchronized operation of the detector^23^. The SIB protects the detector by identifying anomalies and probable hazardous situations in the vacuum chamber and power crates^24^.

### Signal processing

The FPM is equipped with two monolithic $256\,\times \,128$ DEPFET pixels sensors. Each sensor is divided in eight electrically independent octants, composed of $64\,\times \,64$ pixels, that share the single entrance window on the sensor backside. The DEPFET is biased in a common source configuration, and thus the DEPFET source and gate contacts are shared octant-wise, whereas their drain contacts are pixel-wise bump-bond connected to the signal-processing chain of the readout ASIC.

**Figure 1:**
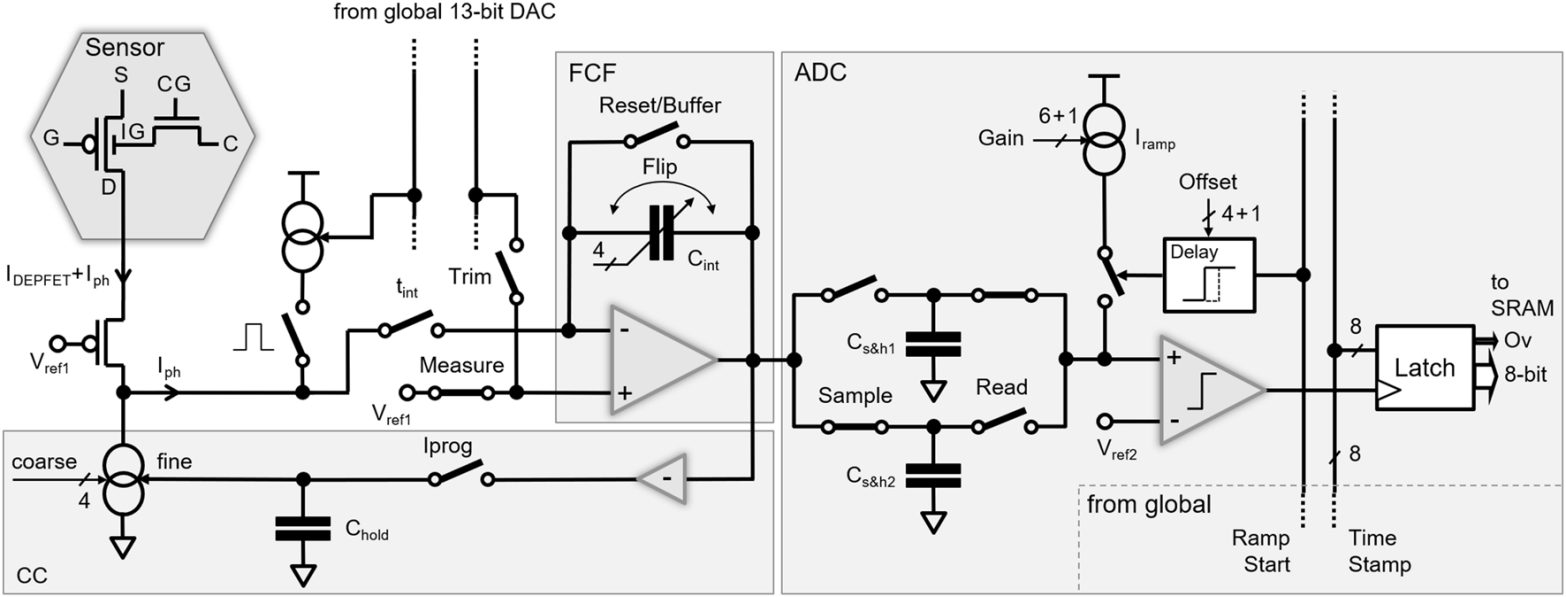
Simplified block diagram of the pixel-level electronics.

**Figure 2:**
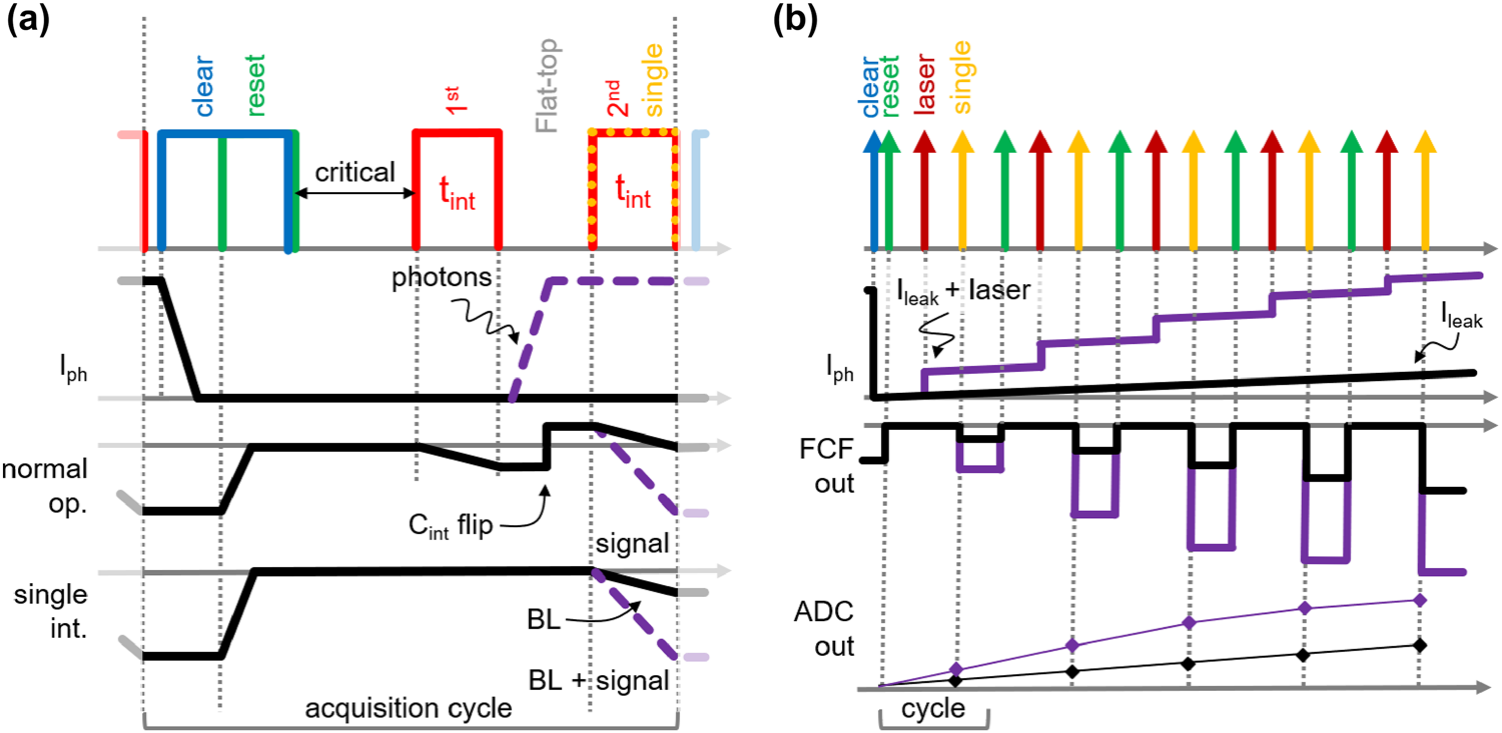
Simplified timing diagram of a single acquisition cycle (**a**) showing the normal and the single integration modes. Special modes in single integration (**b**) are used for the leakage current and the non-linear response characterization.

Figure 1 shows a simplified block diagram of the pixel-level electronics. The front-end circuitry consists of a cascode stage to fix the DEPFET’s drain voltage and a programmable current source (4 bit coarse) to sink the DEPFET quiescent current I_DEPFET_. The fine regulation of the current-compensation (CC) circuit is obtained through an additional analog branch consisting of a capacitor (C_hold_) and a switch (Iprog) connecting the output of the flipped-capacitor filter (FCF)^25^. CC is automatically tuned prior to the arrival of an X-ray bunch train (zero signal). The time-variant filter implements a trapezoidal weighting function and performs an optimal noise filtering at the foreseen readout speed. Its key timing parameters are programmable in 1.44-ns time steps, and four feedback capacitors (C_int_) provide the coarse-gain flexibility to cope with different experimental requirements. The timing diagram for the two operating modes are described in Fig. 2a. In normal operation (normal op.), the filter integrates the DEPFET current twice. In the first phase (1st t_int_) it integrates the residual bias-current (baseline (BL) measurement), and in the second phase (2nd t_int_) it integrates the DEPFET-signal current (BL + signal measurement). Since the feedback capacitance is flipped between the first and the second integration (C_int_ flip), the output of the filter at the end of the second phase is already a measurement of the baseline-subtracted signal. Thanks to its flexibility, the filter can also be programmed in order to integrate only the signal current at the expenses of the BL subtraction (single int.). In any case, the duration of the signal-current integration is controlled by the switch t_int_. As illustrated in Fig. 1, two sample and hold capacitors (C_s&h1,2_) are operated in a double-buffer fashion (alternating sample & read) at the output of the filter. A single-slope Wilkinson-type 9-bit ADC performs the analog-to-digital conversion^26^. The voltage on C_s&h_ is ramped with a programmable current source I_ramp_ (6 bit fine plus double-current bit). The time required for the ramp to reach a reference voltage (V_ref2_) is measured. The 8-bit Gray-coded time stamps are provided column-wise at about $695\,\text{MHz}$, providing a bin size of $720\,\text{ps}$ (dual-edge clocking). The ninth bit (Ov) is generated in-pixel to save routing area. The programmable ramp current allows a fine-tuning of the ADC gain with a resolution of about $2\%$. Additionally, the ADC provides an offset trimming by adjusting the delay between the start of the counter (Ramp Start) and current injection on C_s&h1,2_. The 4-bit controllable delay circuit allows an offset granularity better than $10\%$ of the ADC-bin size. Both trimming capabilities are needed to minimize the pixel-error rate at and below the sensitivity level of one photon per bin^7,26^. For test, trimming and calibration purposes, a global 13-bit digital-to-analog converter (DAC) is available on-chip. The programmable voltage is either provided to a pixel-internal current mirror for pulsed current injection at the negative input of the FCF during measurement (Measure) or provided to its positive input for ADC trimming (Trim) during FCF-buffer mode operation (Reset/Buffer).

A special timing sequence has been defined in order to measure the leakage current flowing into each pixel and to exploit the non-linear response characteristics of the DEPFET device (cf. Fig. 2b). The FCF is set to single integration mode, and the DEPFET internal gate (IG) is cleared only at the beginning of the train. In this way, the collected charge is integrated in the IG over several acquisition cycles and sampled. By means of a pulsed light source synchronized to the system, moreover, it is possible to increase the injected charge every cycle to scan the response of the DEPFET over several acquisition cycles thus relaxing the power constraints of the light source.

### Experimental setup

**Figure 3:**
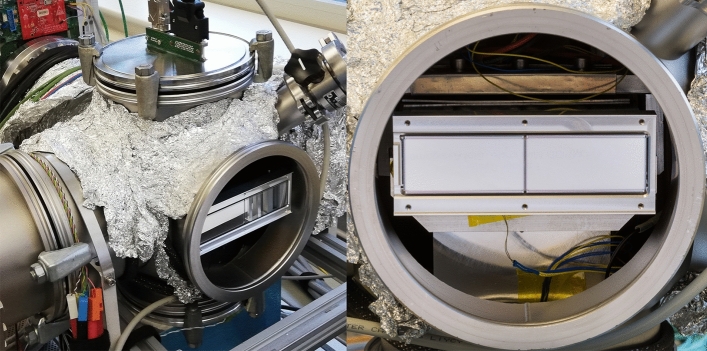
View of the lab-vacuum chamber and particular of a CMOS-DEPFET ladder during the measurement campaign.

The ladder camera head was hosted in a vacuum chamber operated at a pressure of $5\,\times \,10^{-2}\,\text{mbar}$. A Peltier-based cooling system was designed to cope with the in-vacuum power dissipation of the DEPFET ladder resulted up to $18.6\,\text{W}$, which corresponds, for the 1-megapixel detector, to $298\,\text{W}$ in-vacuum and $412\,\text{W}$ considering the outside-vacuum electronics. For comparison, the mini-SDD figures are $9.3\,\text{W}$^7^, $149\,\text{W}$ and $263\,\text{W}$, respectively. The difference in the power dissipation is mainly due to the additional DEPFET-clear electronics (MA and GD) as well as the DEPFET-quiescent current. In the experiments presented here, the cooler kept the sensor temperature constant at around $18\,^{\circ }\text{C}$ to avoid any overheating of the system and to ensure the thermal stability. A flange with a Kapton window was placed in front of the FPM in order to use a radioactive ^55^Fe source outside the vacuum chamber for pixel-wise energy calibration in the linear gain range, or to use an external pulsed laser diode (octant-wise illumination with $100\,\text{ns}$ long pulses at 940-nm emission wavelength) for gain determination in the non-linear range. In both cases, the distance between source and the sensor backside was about $5\,\text{cm}$. Note, that the sensors’ entrance window has a $150\,\text{nm}$ thick light-blocking Al filter requested by the SQS instrument, which might be easily reduced to $30\,\text{nm}$ (mini-SDD camera) increasing the quantum efficiency at low energies. A picture of the vacuum setup is shown in Fig. 3.

In all measurements, the power-cycling mode was used with $600$ μs long active time at a repetition rate of $10\,\text{Hz}$. The ladder was predominantly operated at a frame rate of $2.25\,\text{MHz}$. This operating speed relaxes the operation-critical time gaps between the clear/reset pulses and integration cycle (cf. Fig. 2a: “critical”). In this way, an optimization of levels and timing of source (S), gate (G), clear (C) and clear gate (CG) voltages was possible (Fig. 1). After optimization, the 4.5-MHz and 1.125-MHz modes were successfully operated. An integration time t_int_ = $50\,\text{ns}$ and a clear-pulse width of $50\,\text{ns}$ was used. The clear and clear-gate low levels ($7.5\,\text{V}$ & $4.5\,\text{V}$) as well as the source and gate voltages ($5\,\text{V}$ & $\sim 2.5\,\text{V}$) are switched on $16$ μs prior to the start of a bunch train and switched off at its end. The clear and clear-gate high levels ($20.5\,\text{V}$ & $11\,\text{V}$) are pulsed after each single bunch to clear the collected charge between two acquisition cycles. The IOB controls the operation-critical delay ($7.5\,\text{ns}$ & $5\,\text{ns}$) and width ($50\,\text{ns}$ & $37.5\,\text{ns}$) of the two signals independently. A corresponding timing configuration for 4.5-MHz frame rate was also prepared (t_int_ = $30\,\text{ns}$) and tested after optimization. In order to test the noise performance at lower frame rates, a 1.125-MHz sequence with t_int_ = $300\,\hbox {ns}$ was exploited at different C_int_.

## Results

### DEPFET current

The first operation that must be performed after switching on the sensor is to trim the coarse section of the CC circuit in order to minimize the I_DEPFET_ amount flowing to the FCF. This is done by choosing the coarse setting that allows a voltage on C_hold_, called V_hold_, maximizing the dynamic range of the analog branch. To achieve this, the in-pixel ADC is used to measure V_hold_ before the start of the bunch train at the end of the Iprog phase. Therefore, the ADC gain was beforehand equalized to uniform the response of all the pixels.

**Figure 4:**
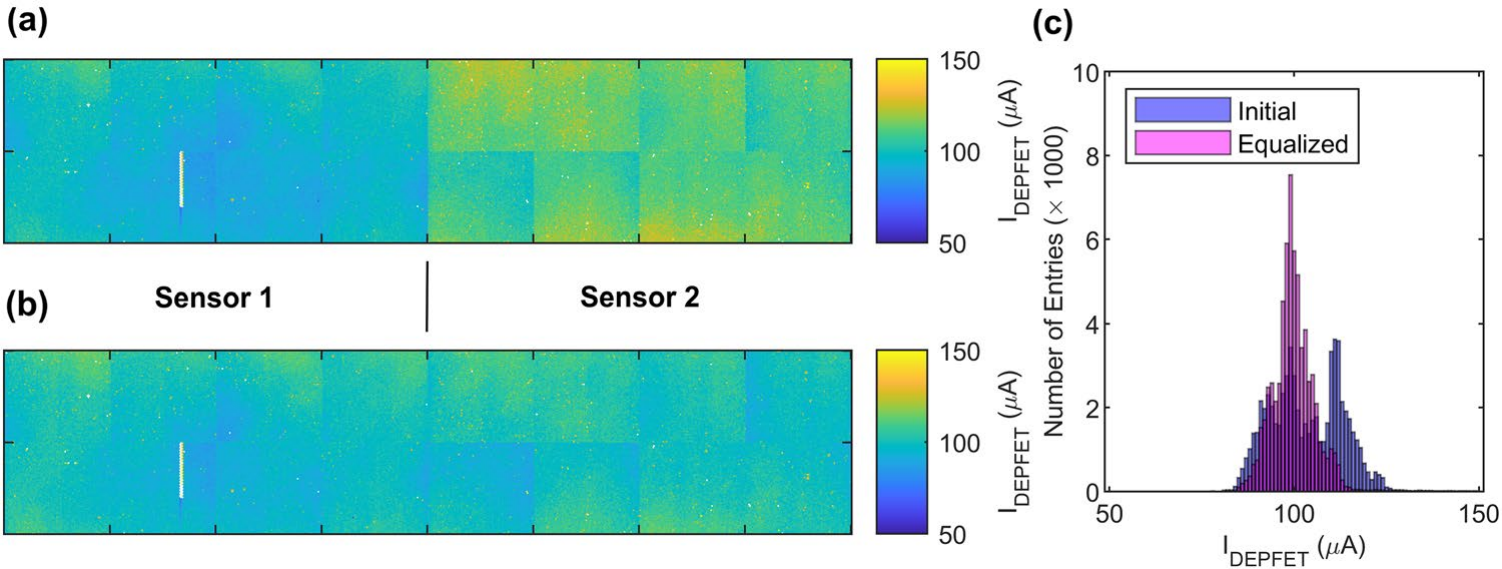
(**a**) DEPFET-quiescent current map of the ladder (512 $\times$ 128 pixels) when both sensor gates are biased at $2.5\,\hbox {V}$. The ticks mark the sensor’s octant separation (64 pixels). Sensor 1 (left) shows an average current slightly below the target value of $100$ μA, while sensor 2 (right) current is more than $10\%$ higher. (**b**) Currents after the equalization. (**c**) I_DEPFET_ distributions before (blue) and after (magenta) the equalization.

The I_DEPFET_ amount flowing into each pixel can be estimated by the knowledge of the CC-coarse setting and the measured V_hold_ thanks to the CC-circuit level simulations of the transfer characteristics. Figure 4a shows the map of the DEPFET-quiescent current for a gate voltage of $2.5\,\hbox {V}$. The electronics and power supply allow to adjust the source and gate voltages independently for the two sensors of the FPM, thus enabling the possibility of adjusting the average sensor-drain currents to obtain a uniform response over the focal plane. This is an important step to also equalize the DEPFET gain in the linear region being proportional to $\sqrt{I_{DEPFET}}$^27^. For this purpose, the CC was trimmed, and the average per-pixel current was computed for three different gate voltages (V_G_). The transfer characteristics (I_DEPFET_ vs. V_G_) allows to find the gate voltages to obtain the desired working point of $100$ μA/pixel. The finally chosen gate voltages were $2.4\,\hbox {V}$ and $2.71\,\hbox {V}$ corresponding to an average I_DEPFET_ of $99$ μA and $101$ μA for sensor 1 and sensor 2, respectively. The source voltage was kept fixed at $5\,\hbox {V}$. The map of the current distribution after equalization, as well as the distribution comparison with the initial biasing condition are shown in Fig. 4b,c. A total number of 89 pixels ($0.14\%$) were excluded from the average computation due to the high leakage currents or defects that prevents the proper operation of the CC circuit. These include the pixels of the two partial columns of sensor 1, where a defect in the middle of the column prevent the operation of the downstream pixels.

### Pixel gain and offset

After equalization of the average quiescent current, the global gain of the system was assessed. An ^55^Fe-radioactive source ($650\,\hbox {MBq}$) was used to irradiate one sensor at a time, achieving an average photon flux on the sensor of about $1.53\,\times \,10^{-4}\,\hbox {photon}/\hbox {pixel}/\hbox {frame}$, which corresponds to $281\,\hbox {photon}/\hbox {pixel}/\hbox {s}$. The radioactive source is an asynchronous source, and therefore the flat-top of the filter weighting function was extended to $500\,\hbox {ns}$ in order to ensure a proper peak-to-shelf ratio in the acquired spectrum (t_int_ = $50\,\hbox {ns}$). This timing variation is not altering the system gain, but increases the effective acquisition-cycle length, and therefore the number of acquirable frames was reduced accordingly to avoid exceeding the electronics power within the active time of $600$ μs. The other timing delays were not changed with respect to the original 2.25-MHz configuration. The lowest C_int_ of the FCF was selected in order to obtain the maximum coarse gain at the given integration time, and the ADC was previously trimmed to its nominal gain. A total of more than 13,000 trains of 400 frames each were acquired, and the histogram data for each pixel extracted. The single pixel spectra were fitted with a simplified fitting function derived from the work of Schlee^28^. In particular, the pedestal peak was fitted with a simple Gaussian function, while the Mn $\hbox {K}_{\upalpha }$ and $\hbox {K}_{\upbeta }$ peak fit comprise a Gaussian function for the peaks and a shelf function to reduce the fit error on the peak position. The resulting average untrimmed gain was $5.07\,\text {ADU}/\,\hbox {keV} \pm 3.62\%$ corresponding to a sensitivity of $197\,\hbox {eV}/\,\text {ADU}$. As the ADC was previously trimmed, the dispersion is mainly caused by the pixel-to-pixel variation in the FCF as well as by process variations in the sensor production.

**Figure 5:**
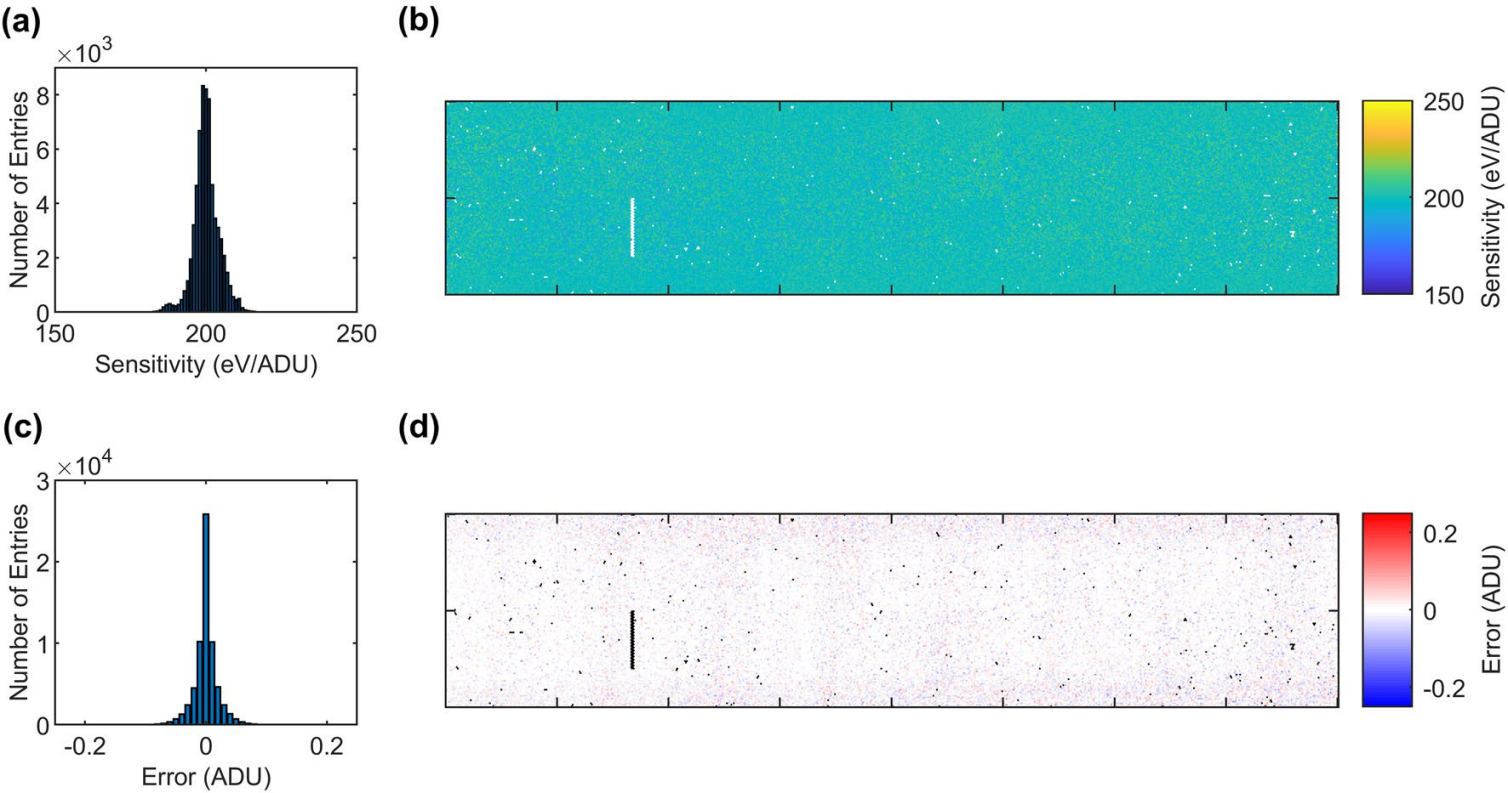
Sensitivity distribution (**a**) and map (**b**) of the system after the offline trimming targeting $200\,\hbox {eV}/\,\text {ADU}$. Offset error distribution (**c**) and map (**d**) after trimming.

To assess the system-trimming capabilities, an offline-gain trimming was performed with a target gain of $5\,\text {ADU}/\,\hbox {keV}$. The ADC gain was measured pixel wise using the in-pixel current injection circuit, and a look-up table of the ADC gain was generated. Then, the gain ratio obtained from the look-up table was used to cross-calibrate each ADC gain, knowing the absolute gain from the radioactive source measurement. Finally, the nearest cross-calibrated ADC gain setting was selected and applied to the FPM. For verification, a new spectrum acquisition was taken and analyzed with the same method described before. A final gain of ${5.00\,\text {ADU}/\,\hbox {keV}} \pm 2.09\%$ was obtained that correspond to a sensitivity of $200\,\hbox {eV}/\,\text {ADU}$, well in line with the expectation and trimming capabilities of the ADC. Less than 400 pixels ($0.6\%$) were excluded from the analysis. These include mainly high-leakage pixels and pixels that show a low sensitivity. A map and a histogram of the sensitivity after trimming are shown in Fig. 5a,b. The ADC-offset trimming capability was also evaluated. For each offset step, a dark acquisition was performed and the pedestal peak fitted. The error with respect to the bin center was computed and the final offset setting chosen as the setting with the minimum offset error. As illustrated in Fig. 5c,d, all pixel offsets were found in the $\pm 0.1\,\text {ADU}$ range with a dispersion of $0.02\,\text {ADU}$. $0.5\%$ of the pixels showed instabilities, which corresponds to the pixels where the gain determination was not possible. These were excluded from the computation.

### DEPFET-leakage current

**Figure 6:**
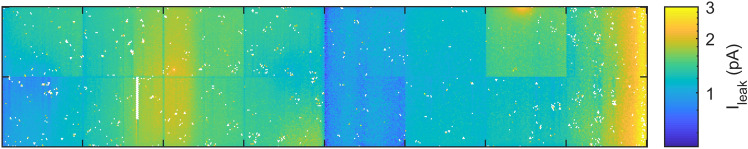
DEPFET-leakage current map showing an average leakage current per pixel of $1.35\,\hbox {pA}$ at room temperature.

In the next step, the pixel-leakage current was measured. The special timing sequence described in “Camera and methods” section (cf. Fig. 2b) was used to measure the leakage-induced charge within the time span of 400 frames. 1000 acquired trains were averaged frame by frame, and the resulting ramp was fitted with a linear regression model. Taking the system timing and the calibration into consideration, the leakage current of each pixel was computed by

$$\begin{aligned} {I_{leak,px} = \frac{\Delta Q}{\Delta T} = \frac{\Delta N}{ \Delta T } \cdot e = \frac{ S }{ G \cdot E_{e-h} } \cdot e,} \end{aligned}$$

where *S*[ADU/s] is the slope, *G*[ADU/eV] the pixel gain, $E_{e-h} = 3.6\,\hbox {eV}$ the energy to create an electron–hole pair in Si, and *e* the electron charge. The average leakage current per pixel was $1.35\,\hbox {pA}$ corresponding to a current density of $2.8\,\hbox {nA}/\hbox {cm}^{2}$. The leakage current map is shown in Fig. 6. Altogether, less than 950 pixels ($1.4\%$) were excluded from the analysis mainly due to the missing gain information or measurement problems caused by the leakage current being too high.

### Gain compression

**Figure 7:**
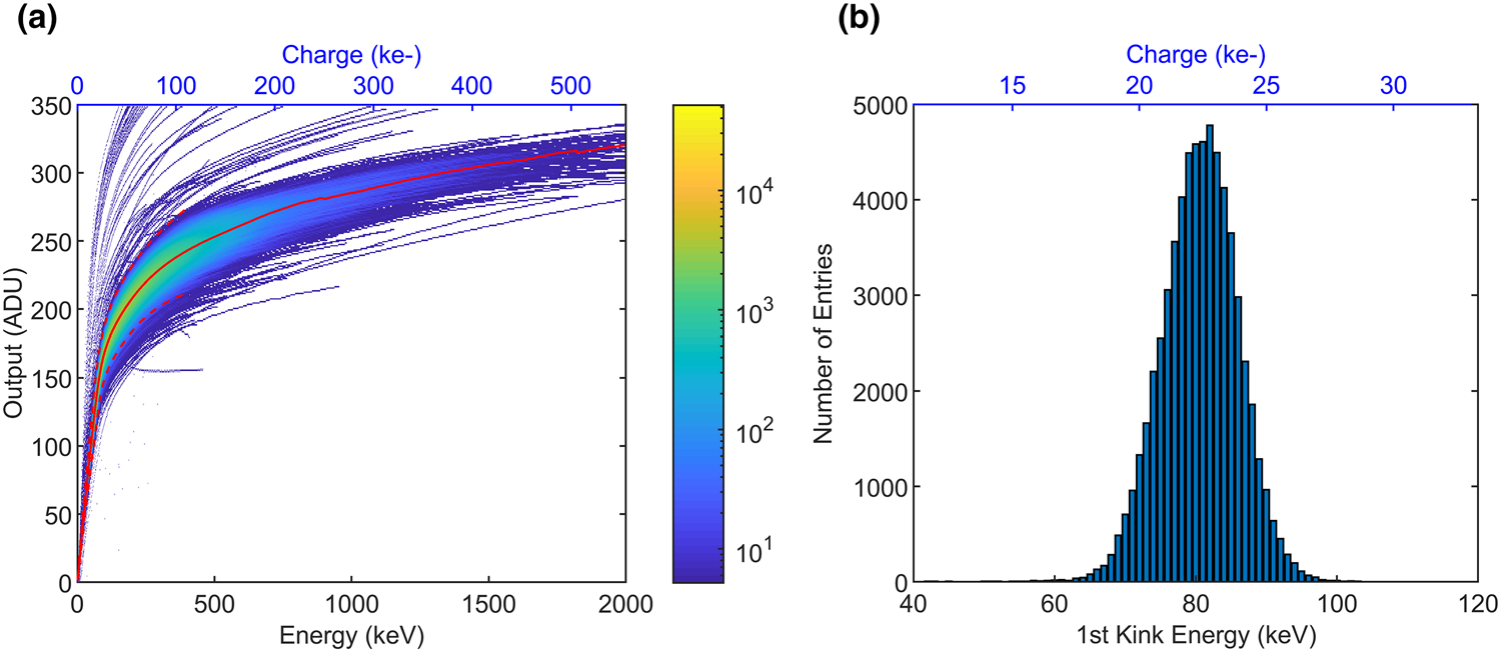
(**a**) Superposition of DEPFET characteristics with the average characteristic (red curve) $\pm 3\sigma$ (dashed curves). (**b**) shows the distribution of energy position of the 1st kink in the non-linear response characteristics.

In another experiment, the non-linear response characteristics was measured in 9-bit ADC mode. Four hundred consecutive $100\,\hbox {ns}$-long laser pulses were used to generate a signal charge in the pixels internal gate. For this purpose, the leakage current measurement timing scheme was used again (cf. Fig. 2b). The integrated charge was measured at a gain suitable to explore the nonlinear characteristics. Therefore, a new ^55^Fe spectrum was acquired at the same gain condition to cross-calibrate the injected charge for each pixel, resulting in a sensitivity of $473 \pm 22\,\hbox {eV}/\,\text {ADU}$. The superposition of the response characteristics of 63,498 pixels ($96.9\%$), as well as the resulting average curve is shown in Fig. 7a. The outliers contain high-leakage pixels, calibration-failed pixels, and pixels with insufficient illumination level. From the average curve it is possible to calculate the gain compression as the ratio between the slope in the linear range versus the slope for energies above $1.5\,\hbox {MeV}$ resulting in a compression ratio of 73.7. Furthermore, it is possible to estimate the dynamic range (DR) by extrapolating the average response curve above the maximum measured value. Taking the gain and saturation levels of the front-end electronics into account, the average DR is $2.13\,\hbox {MeV}$ corresponding to $591\,\hbox {k}\text {e}^{-}$ or 4500 photons of an energy of $473\,\hbox {eV}$ at one photon per bin resolution.

The minimum of the 2nd derivative of the response characteristics represents the position of the first DEPFET gain change (1st kink). It was computed and an average kink energy of $80.61 \pm 5.44\,\hbox {keV}$ was found. Figure 7b displays the dispersion of the 1st kink energy.

### Equivalent noise charge

**Figure 8:**
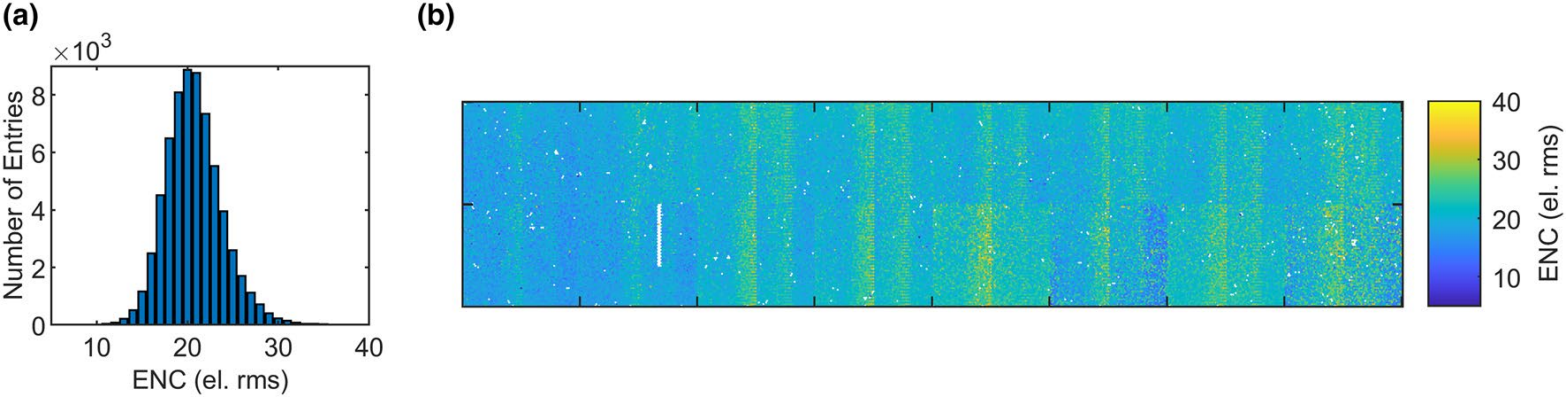
Distribution (**a**) and map (**b**) of the equivalent noise charge.

Finally, the ENC was measured in the same gain configuration as for the previous measurements but without the increased flat-top time used for the ^55^Fe measurement (flat-top time $43.2\,\hbox {ns}$). Thanks to the offset-trimming capabilities of the system, we used an ADC-bin edge as a knife edge to measure the pedestal width. First, the pixel-offset step size was determined using the method described by Hansen et al.^26^. For all possible 16 offset steps, a ramp was generated, and a signal was acquired using the DEPFET-leakage current following the same technique as for its measurement. The 16 ramps were fitted with a linear regression. The slope of the subsequent linear regression of the intercepts versus the offset setting determines the offset average step size of $133.8 \pm 21.3\,\hbox {ps}$. The pedestal width was then measured acquiring a dark run of 250 trains for each offset setting. The number of counts above a reference ADC bin were extracted, and the resulting cumulative distribution function was fitted to extract the pedestal width. The ENC was then computed taking the calibrated gain of $5.00\,\text {ADU}/\hbox {keV}$ into account. At an integration time of $50\,\hbox {ns}$, an average ENC of $20.7 \pm 3.1\,\text {e}^{-}\text {rms}$ was measured. Figure 8 shows the histogram and map of the noise.

### Spectral performance and dynamic range

During the above-mentioned test campaign using the first prototype ladder, a second ladder was tested to confirm the results obtained with the first prototype and to further investigate the noise and dynamic range performances of the system at different ASIC operating conditions. The DEPFET bias voltages and timing were kept fixed, as well as the target quiescent current of $100$ μA. For each operating frequency, a spectrum acquisition and noise analysis have been conducted using the same methods as described above for the first prototype. In addition, the DR for each pixel was extrapolated. The DR determination method for the DSSC imager is described by Porro^29^. First, the ASIC DR was determined by subtracting the saturation level of each pixel from the pedestal centroid position. Then, the non-linear response, that is independent of the ASIC gain configuration, was taken into account. Due to the lack of data at high injection levels for all the ladder pixels, the average characteristics on the 165 pixels showing injection up to $1.5\,\hbox {MeV}$ was used. In order to cover the required energy range to reach the ASIC saturation, the response was extrapolated by means of a linear regression of the last $100\,\hbox {keV}$ to reach the saturation limit of the ASIC.

In a first experiment, the same conditions of the first ladder under test were applied. At the operating frequency of $2.25\,\hbox {MHz}$ and an integration time of $50\,\hbox {ns}$, the same low noise performance was obtained. For the trimmed and calibrated gain of ${5.05\,\text {ADU}/\hbox {keV} \pm 2.2\%}$ the resulting ENC was $18.5 \pm 2.7\,\text {e}^{-}\text {rms}$. The average DR was $19\,\hbox {k}\text {e}^{-}$. The same analysis was performed at two lower gain settings by increasing C_int_ of the FCF, but skipping the gain trimming. At a gain of ${2.2\,\text {ADU}/\hbox {keV} \pm 4.4\%}$ and ${1.63\,\text {ADU}/\hbox {keV} \pm 5.3\%}$, an ENC of $26.6 \pm 6.4\,\text {e}^{-}\text {rms}$ and $34.4 \pm 9.5\,\text {e}^{-}\text {rms}$, and a DR of $489\,\hbox {k}\text {e}^{-}$ and $1.345\,\hbox {M}\text {e}^{-}$ can be obtained, respectively.

Another frequency of interest for the system is $1.125\,\hbox {MHz}$ that corresponds to the maximum bunch-pattern frequency currently used in the mini-SDD camera experiments. This represents also the maximum bunch frequency foreseen in the next EuXFEL continuous wave upgrade in the so-called long-pulse mode^30^. This mode allows to increase the integration time up to $300\,\hbox {ns}$ for the best noise performances of the DSSC. In two experiments, the FCF feedback capacitor value was changed in order to explore the performance at the low gain, for highest DR, and at high gain, for best noise performance. The lowest gain obtained was ${2.5\,\text {ADU}/\hbox {keV} \pm 5.5\%}$ and an ENC of $20.14 \pm 5.6\,\text {e}^{-}\text {rms}$. At high gain mode, we reached ${13.4\,\text {ADU}/\hbox {keV} \pm 2.2\%}$ and an ENC of $10.5 \pm 2.1\,\text {e}^{-}\text {rms}$. The DR were $165\,\hbox {k}\text {e}^{-}$ and $7\,\hbox {k}\text {e}^{-}$ at low and high gain, respectively.

**Figure 9:**
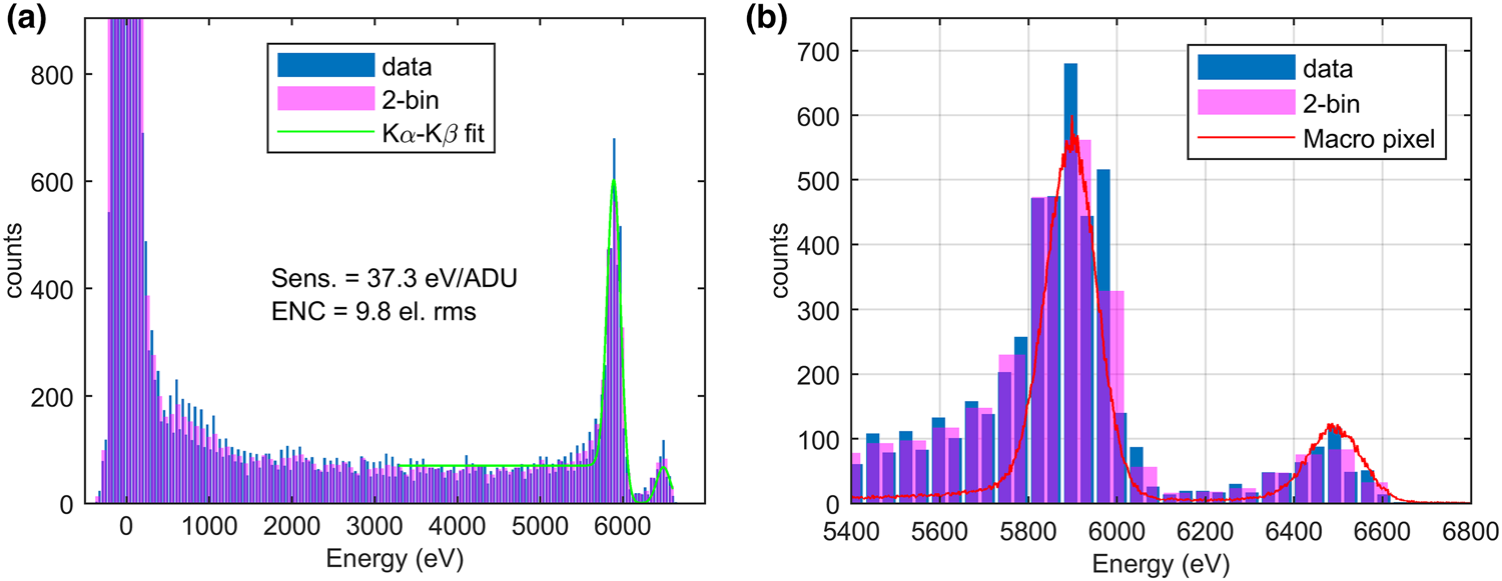
^55^Fe spectrum (**a**) obtained at $1.125\,\hbox {MHz}$ showing the spectroscopic capabilities of the sensor at $18\,^{\circ }\hbox {C}$ and 300-ns integration time and increased flat-top time of $500\,\hbox {ns}$. (**b**) Zoom in the Mg peak region and a reference spectrum of a macro pixel from an early development stage sample of the CMOS-DEPFET fabrication measured at $-50\,^{\circ }\hbox {C}$ and ${3}$-μs shaping time (red curve).

To explore the spectroscopic capabilities of the system, we further increased the overall gain near to the limits of the system by doubling the ADC gain, but keeping margin for the fine gain trimming. A final gain of ${26.8\,\text {ADU}/\hbox {keV} \pm 2.5\%}$ was leading to the best noise performance of $9.8 \pm 1.4\,\text {e}^{-}\text {rms}$. Figure 9 shows a representative ^55^Fe spectrum of a sample pixel. For comparison a spectrum of a macro pixel from an early development stage sample of the CMOS-DEPFET fabrication, a single DEPFET structure surrounded by SDD-type drift rings, is shown. The measurement was performed at $-50\,^{\circ }\hbox {C}$ and ${3}$-μs shaping time^19^. A re-binned histogram, where two bins are merged in order to mitigate the DNL effects^31^, is also displayed (cf. 2-bin). The DNL is mainly caused by the duty-cycle spread in the time-stamp distribution over the matrix. The average DR in this configuration is $5\,\hbox {k}\text {e}^{-}$. Finally, we evaluated the performance at the maximum operating frequency of $4.5\,\hbox {MHz}$. This operating frequency is crucial for the timing sequence and does not allow any freedom in the choice of the parameters. In particular, an integration time of $30\,\hbox {ns}$ was used in order to keep the DEPFET-timing scheme unchanged. The ADC gain was kept fixed, but the change in the integration time reduced the front-end gain by about $40\%$. An average gain of ${3.12\,\text {ADU}/\hbox {keV} \pm 3.65\%}$ was finally obtained, and the ENC analysis reports a noise of $25.5 \pm 5.3\,\text {e}^{-}\text {rms}$ confirming the simulated performances of the system. The estimated average DR was $26\,\hbox {k}\text {e}^{-}$. All the results are summarized in Table 1.

**Table 1:** Summary of the results.

Frame rate ($\hbox {MHz}$)	t_int_ ($\hbox {ns}$)	Filter C_int_ ($\hbox {pF}$)	ADC coarse gain	Gain ($\,\text {ADU}/\hbox {keV}$) (%)	ENC ($\text {e}^{-}\text {rms}$)	Dyn. range ($\hbox {k}\text {e}^{-}$)
4.5	30	1	1$\times$	3.12 ± 3.65	25.5 ± 5.3	26
2.25	50*	1	1$\times$	5.05 ± 2.2	18.5 ± 2.7	19
		2.5	1$\times$	2.2 ± 4.4	26.6 ± 6.4	489
		3.4	1$\times$	1.63 ± 5.3	34.4 ± 9.5	1345
1.125	300	1	2$\times$	26.8 ± 2.5	9.8 ± 1.4	5
		1	1$\times$	13.4 ± 2.2	10.5 ± 2.1	7
		13.8	1$\times$	2.5 ± 5.5	20.14 ± 5.6	165

*The integration time for the $2.25\,\hbox {MHz}$ operation was not maximized and can be increased, if needed, up to about $100\,\hbox {ns}$.

## Discussion and perspective

**Figure 10:**
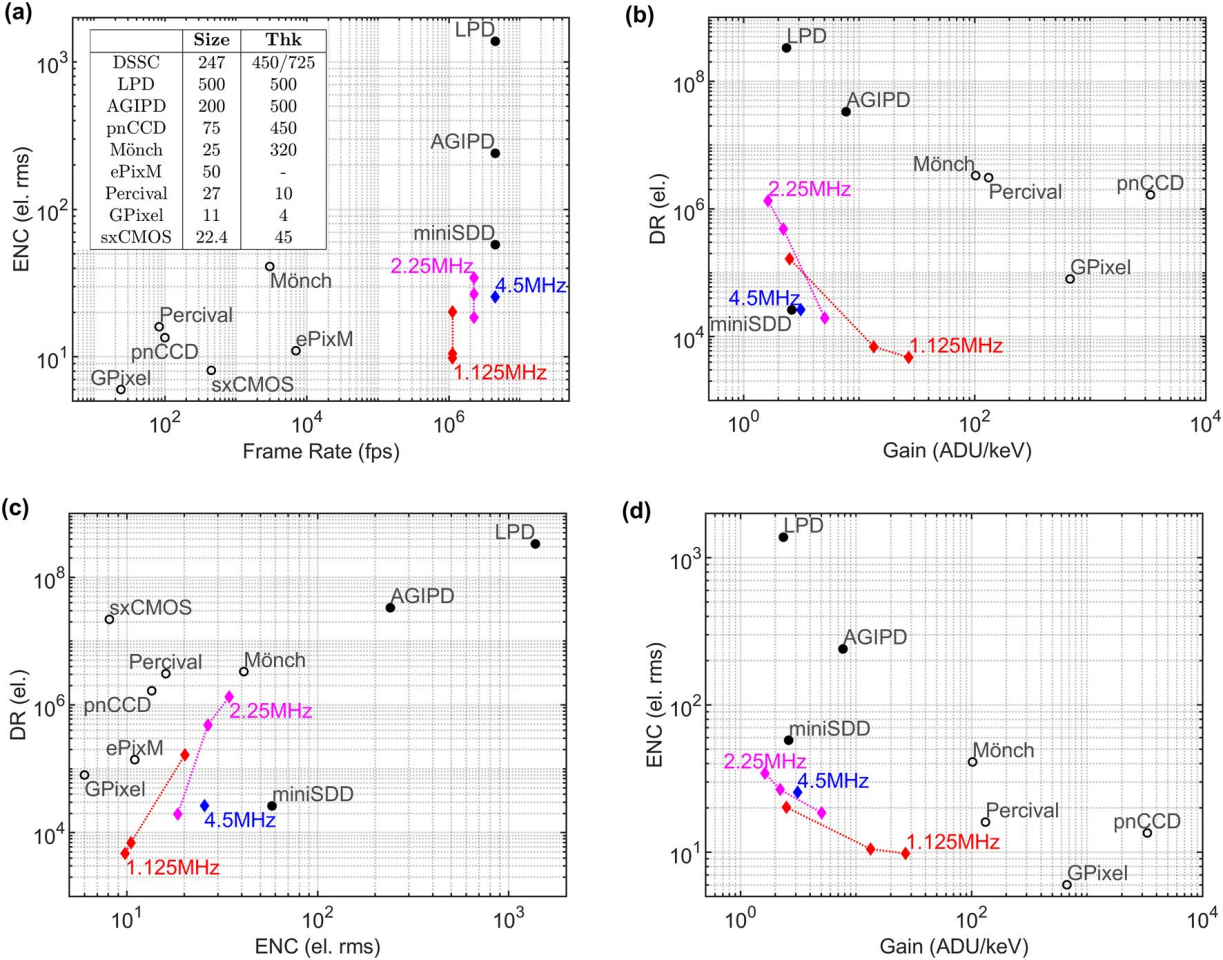
Comparison of key figures for different detectors with respect to the CMOS-DEPFET detector (colored symbols). (**a**) Noise versus peak frame rate, together with an overview table on the pixel size and active thickness (Thk), both in μm. (**b**) Dynamic range versus gain, where gain data for ePixM and sxCMOS is not available. (**c**) Dynamic range versus Noise, and (**d**) Noise versus gain, where again the gain data for ePixM and sxCMOS is not available.

A summary of the CMOS-DEPFET ladder performance is presented in Fig. 10 together with the state-of-the-art detector systems aforementioned with the data available in literature^2,4,12–17^.

As can be seen in Fig. 10a, the ENC achieved with the prototype ladders is comparable to the soft X-ray detectors at all the operating frequencies. The outstanding noise figure of $9.8\,\text {e}^{-}\text {rms}$ is obtained at the peak frame rate of $1.125\,\hbox {MHz}$. The in-pixel electronics is capable to sustain a continuous MHz-frame rate with excellent noise performance. Compared to the detector in operation at EuXFEL, DSSC is placed in the unique position being able to cope with the XFEL bunch pattern at the ENC levels of the soft X-ray detectors.

DSSC features generally a lower gain in contrast to all the detector alternatives. This is mainly associated to the lower ADC resolution and thus the need to adjust the detector sensitivity to the incident photon energy in order to maximize the performance. The CMOS-DEPFET first gain compression kink is at around $80\,\hbox {keV}$, and therefore the DR reaches the level of other soft X-ray detectors when the ASIC is operated at lower gains (Fig. 10b). Depending on the final user application, the single-photon resolution or the DR can be optimized (Fig. 10c). Lowering the photon energy, thus increasing the gain to achieve single photon resolution, lowers the achievable DR (Fig. 10d).

The DEPFET-equipped ladder showed its unique features and opens new possibilities in the X-ray imaging at megahertz frame rate. The front-end architecture together with the CMOS-DEPFET sensor proved to be an excellent base for further developments in the low-noise soft X-ray detectors at ultimate readout speeds. The DEPFET-dynamic range can be further tailored to the application by modifying the shape of non-linear response characteristics, e.g. moving the gain kinks to lower energies. Finally, the possibility to scale the pixel size, together with the usage of smaller technology node for the readout ASICs, will allow one to build higher spatial resolution cameras with megahertz readout speed, high dynamic range and ultimate single-photon sensitivity. With a proper power management, moreover, CW operation at higher bunch rates is also possible. This would make DEPFET and DSSC attractive for many facilities beyond the EuXFEL.

## Summary

Two DSSC ladder protoypes equipped with CMOS-DEPFET sensors were successfully operated and characterized. The first laboratory test campaign confirms the functionality of the electronics and readout ASIC, and shows the excellent performances achievable with the new sensors. The gain and noise figures have been experimentally evaluated, and a method to scan the non-linear response has been proposed. This allowed the estimation of the dynamic range. The protoypes were operated at different readout speeds to explore the capabilities of the system. The demonstrated performance fulfill the requirements at the EuXFEL soft X-ray instruments, and we started the series production to build the full camera.

## Data Availability

The data presented in this study are available upon reasonable request to the corresponding author.
